# Draft genome sequence of *Listeria monocytogenes* BDSA strain isolated from ready-to-eat chicken product in Dhaka, Bangladesh

**DOI:** 10.1128/mra.00956-24

**Published:** 2025-01-13

**Authors:** Sunjida Afrin, Michael Netherland, Halima Habib, Nur Hasan, Sangita Ahmed

**Affiliations:** 1 Department of Microbiology, University of Dhaka, Dhaka, Bangladesh; 2 EzBiome Inc., Gaithersburg, USA; University of Maryland School of Medicine, Baltimore, Maryland, USA

**Keywords:** *Listeria monocytogenes*, foodborne pathogen, draft genome, Bangladesh

## Abstract

We present the genome of *Listeria monocytogenes* BDSA isolated from ready-to-eat (RTE) meat collected in Dhaka, Bangladesh. The genome displays the Listeria pathogenicity island 1 and virulence, stress response, and antimicrobial resistance genes. It was phylogenetically classified as ST7, and clustered with serotype 1/2a belonging to lineage II.

## ANNOUNCEMENT

*Listeria monocytogenes* is a leading foodborne pathogen that causes listeriosis, with a high mortality rate in vulnerable populations like immunocompromised individuals, pregnant women, neonates, and the elderly (1). In Bangladesh, *L. monocytogenes* is commonly found in environment and food products (2–7), yet there is limited data regarding the genomic characteristics of strains circulating in the country. This study reports the draft genome sequence of an *L. monocytogenes* strain BDSA isolated from a ready-to-eat chicken product in Bangladesh (5).

The *L. monocytogenes* BDSA isolate was maintained in glycerol stock at −20°C and was revived on Tryptic Soy agar at 37°C for 24 hours. Using the Monarch Genomic DNA purification kit (Biolabs, New England), genomic DNA (gDNA) was extracted from an overnight Tryptic Soy broth culture (incubated at 37°C and 120 rpm) using the manufacturer’s protocol. The sequencing was performed in EzBiome using the Illumina NextSeq 2000 platform (Illumina, San Diego, CA, USA) using the paired-end 150 bp run. DNA libraries were prepared using NEBNext Ultra II FS DNA Library Prep Kit for Illumina (8), following the manufacturer’s instructions for fragmentation, ends repair, 3′ adenylation, and adapter ligation, PCR amplification of gDNA. After clean-up, the libraries were quantified and qualified using the D1000 ScreenTape on an Agilent 2200 TapeStation, normalized, and pooled for multiplexed sequencing using the paired-end 150 bp run format.

The raw sequence data were quality checked using FastQC v0.12.0 (9), followed by quality trimming using Trimmomatic v0.39 (10) and *de novo* assembly of contigs using SPAdes v3.15.5 assembler (11). Any potential contaminations and assembly quality were checked by CheckM v1.2.2 (12) and QUAST v5.2 (13), respectively. The assemblies were taxonomically assessed using EzBiome Bioinformatics App (www.ezbiome.app). For typing, BIGSdb-Pasteur was used (14). NCBI Prokaryotic Genome Annotation Pipeline (PGAP) v6.8 and RAST were employed for annotation and subsystem analysis, respectively (15, 16). VFDB and MegaRes databases from ABRicate (17) were used for the characterization of antimicrobial resistance and virulence gene carriage. Phylogenetic and comparative genomic analyses were performed using kSNP4 (18) and visualized in iTOL v6.9 (19). Default parameters were used for all software unless otherwise specified.

**Fig 1:**
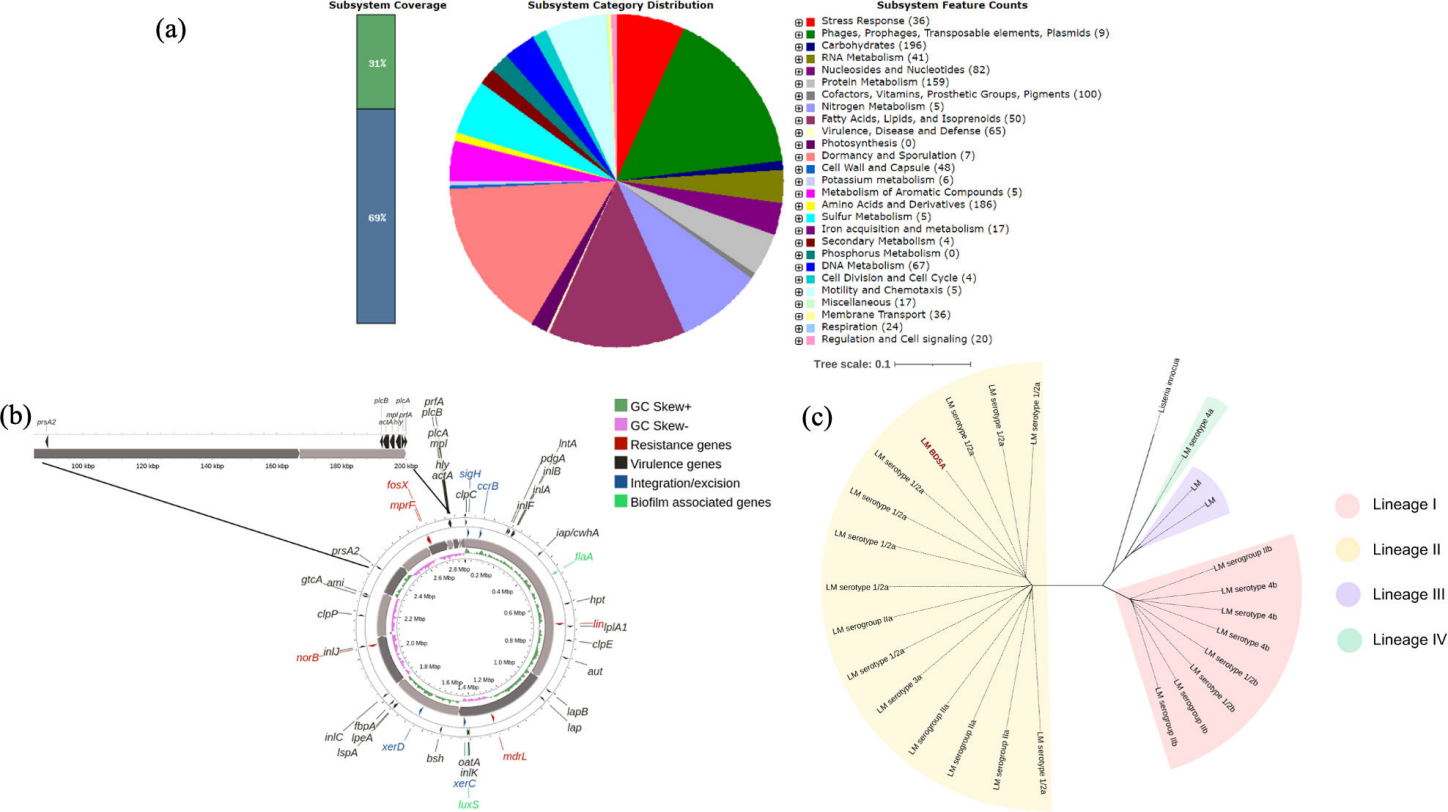
(**a**) Subsystem statistics of *L. monocytogenes* BDSA. (**b**) Genome map of *L. monocytogenes* BDSA highlighting the virulence and antimicrobial resistance genes. The LIPI-1 is shown separately on the upper left side. (**c**) Core-genome SNP-based (cgSNP) unrooted maximum likelihood phylogenetic tree illustrating the homology between the genome of *L. monocytogenes* BDSA (LM BDSA) and representatives of different phylogenetic lineages retrieved from NCBI. The genome sequence of *L. innocua* was used as the outgroup.

The sequencing yielded a total of 6,83,118 bp paired-end sequences and following trimming, 6,53,136 bp sequences were utilized to construct the draft genome (2,843,726 bp) with 99.45% completeness. The GC content was 37.97% and the number of base pairs in the N50 was 475,872 bp (Table 1). No contamination or strain heterogeneity was observed within the assembly sequences. A total of 267 subsystems including stress response, dormancy and sporulation, and metabolism of aromatic compounds were identified (Fig. 1a). The genome revealed the presence of the Listeria Pathogenic Island 1 (LIPI-1), along with 31 major virulence genes and *prsA2*, a positive regulatory factor for the virulence mechanism and pathogenicity (Fig. 1b). Two biofilm forming genes, five antimicrobial resistance genes and Integration/excision genes, were also found. The MLST profile of *L. monocytogenes* BDSA classified it into ST7, while the core genome SNP tree revealed a close relationship with serotype 1/2a belonging to lineage II (Fig. 1c).

**TABLE 1:** Assembly statistics and important genomic features of the *L. monocytogenes* BDSA from ready-to-eat chicken sample in Bangladesh

Feature	Data for *L. monocytogenes* BDSA
Sample source	Ready-to-eat chicken product
Genome size (bp)	2,843,726
Number of contigs	31
Genome coverage (×)	27
GC content (%)	37.97
Largest contig (bp)	983,116
Smallest contig (bp)	201
Average contig length (bp)	91,733
N50 (bp)	475,872
N75 (bp)	228,985
L50 (bp)	2
Number of subsystems	267
Total genes	2,856
Total CDS	2,775
RNA	81
Antibiotic resistance gene	6
Virulence gene	31
Accession No.	JAXGGK000000000

## Data Availability

The draft genome sequence data wasdata were deposited to the National Center for Biotechnology Information (NCBI) under BioProject accession number PRJNA1046657; for assembled genome BioSample accession number JAXGGK000000000; for raw sequence reads SRR27229455. Annotation utilizing NCBI Prokaryotic Genome Annotation Pipeline v.6.8 was used and L. monocytogenes sequence (GCF_034661155.1) was used as a reference.
